# The impact of left-behind experience on the mental health and marital satisfaction

**DOI:** 10.3389/fpsyg.2025.1603281

**Published:** 2025-05-27

**Authors:** Jichao Zheng, Lei Yan

**Affiliations:** ^1^Department of Economics Research, Anhui Academy of Social Sciences, Hefei, China; ^2^School of Marxism, Jiangsu University of Science and Technology, Zhenjiang, China

**Keywords:** left-behind experience, mental health, depression, marital satisfaction, migration

## Abstract

**Background:**

China has a large number of people who migrate internally to work outside their hometown, and at the same time, tens of millions of left-behind spouses stay home alone to take care of their families, so it is important to study the psychological health and marital satisfaction of left-behind spouses.

**Method:**

We used the China Family Panel Studies (CFPS) data 2022 and 2020, which included 8,096 married respondents, 437 of whom have left-behind experiences. Linear regression was used to examine the relationship among left-behind experience, depressive symptoms, and marital satisfaction. We also ran regressions on male and female samples and urban and rural samples to find the heterogeneous effects of the experience of staying behind.

**Results:**

Short-term and previous left-behind experiences had no significant effect on the mental health of the left-behind spouses. Only long-term left-behind experience had a significant negative impact, resulting in an increase in the depression value by 1.16. Long-term left-behind experience also negatively affected the marital satisfaction of the left-behind spouses, decreasing the marital satisfaction value by 0.26.

**Conclusion:**

Long-term left-behind experience is detrimental to the psychological health and marital satisfaction of the spouses left behind, and efforts should be made to reduce the separation of couples due to going out to work and to promote local employment. At the same time, rural left-behind women are a special vulnerable group, and efforts should be made to improve their mental health and provide them with more social support and psychological intervention.

## 1 Introduction

Economic disparities between countries and between regions within countries have led to an increasing prevalence of migration, with a growing number of left-behind spouses as family members fail to migrate in tandem. In Asia, many people are leaving their former areas of residence to work outside the home in search of higher incomes (Nguyen et al., 2006). For example, 27% of the Indian population can be recognized as “migrants” according to the fact that they no longer live in the town or village where they were born (Desai and Banerji, 2008). China is no exception; over the past few decades, Chinese society has evolved from a traditionally low-mobility society to one in which there are rural migrants everywhere (Fan et al., 2011). Some studies have estimated the number of rural–urban migrants in China as high as 140 million (Hu et al., 2008). As many couples do not migrate at the same time to work outside the home, a large number of spouses are left behind, especially women. A study in China estimated that the number of left-behind wives between 20 and 59 was 30.44 million in 2015 (Duan et al., 2017). Another study estimates that 47 million wives are left behind in rural areas (Yi et al., 2014). The exact number may not be known, but there is no doubt that the number of left-behind spouses is very high.

Separation from family can affect a person’s mental health, and there has been a significant amount of research focusing on the impact of migrant employment on migrants’ mental health, but the findings have been inconsistent; for example, some studies have found that migrant workers have high levels of depression (Qiu et al., 2011). Some studies suggest that healthier people will choose to migrate due to the salmon effect (Lu and Qin, 2014). As a result, some studies have found that migrant workers have a small advantage in mental health (Li et al., 2014). There are also many studies that focus on the impact of parental migration on the mental health of the children left behind (Gao et al., 2010; Zhao et al., 2018). There is also a good deal of literature on the mental health of stay-at-home wives (Hussain et al., 2023; Bhirtyal and Wasti, 2021). However, to date, the literature on the impact of left-behind experience on the mental health of left-behind spouses based on Chinese data is still insufficient, and the existing literature rarely distinguishes between the length of the left-behind experience of left-behind spouses or tests for heterogeneity by gender or urban/rural area.

On the other hand, the left-behind experience will affect the marital satisfaction of left-behind spouses. Most people will get married, and marriage quality is critical to one’s happiness (French et al., 2019). Separated married couples are unable to reap the benefits of economies of scale, such as sharing bills (Chen et al., 2015). It has been suggested that physical separation of spouses due to migration is similar to the dissolution of a marriage (Tong et al., 2019).Separation can impact the quality of marriage; for example, due to the absence of spouses from home, there is a lack of direct channels of communication between spouses, and process communication and content are associated with marital satisfaction (Boland and Follingstad, 1987). In addition, as one partner migrates away from home to take up a job, the pressure on the stay-at-home spouse to care for the family increases. And stress threatens marital satisfaction (Randall and Bodenmann, 2009). As a result, one study found that migrant couples typically have higher divorce rates than non-immigrant couples (Caarls et al., 2015).

After combing through the existing literature, we found that, as a whole, there is not much literature focusing on the mental health and marital satisfaction of left-behind spouses, and because the duration of the left-behind experience varies, it is even rarer to see literature studying the heterogeneous effects of the long-term left-behind experience and the short-term left-behind experience on the mental health of the left-behind spouses. Therefore, this paper will focus on the impact of factors such as short-term left-behind experience and long-term left-behind experience on the mental health and marital satisfaction of left-behind spouses. At the same time, the article will further analyze the gender and urban–rural differences in the impact of the left-behind experience to make a modest contribution to this gap area.

## 2 Materials and methodology

### 2.1 Data sources

The data used in our study come from the China Family Panel Studies (CFPS). This survey aims to reflect social, economic, demographic, educational, and health changes in China and to provide a database for academic research and public policy analysis. The CFPS is a nationwide, large-scale social tracking survey that focuses on the economic and non-economic well-being of Chinese residents and various research topics, including economic activities, educational outcomes, family relationships and family dynamics, population migration, health, etc. (The Institute of Social Science Surveys of Peking University, 2025). The CFPS project was implemented by the Institute of Social Science Surveys (ISSS) of Peking University and approved by the Biomedical Ethics Committee of Peking University under the ethical review approval number IRB00001052-14010 (The Institute of Social Science Surveys of Peking University, 2022).

We use data from the CFPS 2022 and 2020 surveys. In 2022, 698 interviewers participated in the CFPS interviews, with the implementation period beginning in May 2022 and ending at the end of 2022. The final questionnaire covered more than 23,000 interviewed family units and generated nearly 58,000 individual questionnaires. The CFPS public data we used removed all privacy variables that could identify individuals to protect respondent privacy information (The Institute of Social Science Surveys of Peking University, 2024). Because we are studying the impact of the left-behind experience on left-behind spouses, we limit our sample to married respondents. Considering that the legal age of marriage in China is 22 for men and 20 for women, we set the lower age limit of the sample in 2022 to 24 for men and 22 for women. Most spouses leave home because they work outside the home, so we focus on respondents who are within the working age. Since the current maximum retirement age in China is 60, we set the upper age limit for the 2022 sample at 60. And all samples are married. Following the criteria of being married and within the working age range, we screened respondents who did not have missing values on all explanatory variables and covariates during the two surveys in 2022 and 2020, resulting in 8,096 respondents. In addition, 437 of these 8,096 respondents had either a long or short experience of staying behind.

### 2.2 Dependent variables

The two dependent variables we focused on were depressive symptoms and marital satisfaction. Major depression is a common disorder (Kessler and Bromet, 2013). Symptoms of depression include depressed mood and loss of interest or pleasure (Paykel, 2008). In the general population, depression is primarily screened for through short self-report scales (Radloff, 1977). In the CFPS, depression values are represented by the CES-D-8, a scale that has been shown to have high internal consistency (Liu et al., 2023). The questionnaire asked respondents how often the following eight feelings or behaviors had occurred in the past week. There were eight questions, six of which were positively scored, including “I feel depressed.” “I find it difficult to do anything.” “I have trouble sleeping.” “I feel lonely.” “I feel sad and upset.” and “I feel like I cannot go on with my life.” Reverse scoring questions included “I feel pleased.” and “I live a happy life.” The Cronbach’s alpha values for the eight-item depression scale used in our study were 0.77 and 0.76 for 2022 and 2020, respectively. Based on relevant literature studies, this alpha value is acceptable and can be used for empirical analysis (George and Mallery, 2003).

Each question had four options: 1. hardly ever (less than a day), 2. some days (1–2 days), 3. often (3–4 days), and 4. most days (5–7 days). For the positively scored questions, each option was scored 1–4, and for the reverse scored questions, each option was scored 4–1. We summarized the answers to the eight questions above and constructed a continuous variable ranging from 8 to 32, with larger values indicating more severe depression.

The marital satisfaction variable uses respondents’ subjective perceptions of their marital life. We constructed a marital satisfaction variable based on the CFPS questionnaire, “Overall, how satisfied are you with your life in your current marriage? (1 is very dissatisfied, 5 is very satisfied).” Using the answers to the above questions as a basis, we constructed marital satisfaction variables, taking values from 1 to 5. The higher the value, the higher the marital satisfaction.

### 2.3 Explanatory variables

The central explanatory variable we focus on is the respondents’ living status with their spouses. This refers to the residential arrangement of the respondent and their spouse. The questionnaire includes a question about whether the respondents’ spouses lived at home. For those whose spouse did not live at home, the questionnaire further asked the reason for leaving the home, ‘Why is he/she not living in this home?’ The questionnaire lists several options, including going to school, seeing a doctor, working outside the home, etc. We consider respondents who chose “spouse works outside the home” as left-behind spouses. The term “not living at home” in the questionnaire refers to not living at home for a more extended period, e.g., more than 3 months. In the Chinese culture, choosing to work outside the home usually means going far away from home, and those who work outside the home seldom go home. For many families, the Chinese New Year reunion is an annual opportunity for family unity (Li, 2014). With regard to the duration of the left-behind experience, one study interpreted the left-behind experience as experiencing at least 6 months of left-behind (Aryal et al., 2020). Another study categorized the left-behind experience into three time periods according to duration: less than or equal to 1 year, 1 to 3 years, and more than 3 years (Lu, 2012). As for our study, because the interval between the two surveys was 2 years.

We designed the left-behind variable based on the respondents’ answers to the above questions. Those respondents who chose their spouse to work away from home in the 2020 or 2022 questionnaire were considered to be respondents in a left-behind status in that year, while those who explicitly stated that their spouse was living at home in the 2020 or 2022 questionnaire were considered to be in a non-left-behind status. We consider respondents whose spouses worked outside the home in both 2020 and 2022 as respondents with long-term left-behind experience, respondents whose spouses worked outside the home only in 2022 as respondents with short-term left-behind experience, respondents whose spouses worked outside the home in 2020 but returned to the home in 2022 as respondents with previous left-behind experience, and respondents whose spouses lived at home in both 2020 and 2022 as the control group.

### 2.4 Covariates

As with most studies (Remes et al., 2021; Kowal et al., 2021), we categorize covariates into three main groups. The first group is demographic background factors, including gender, rural, age, and education. These are important factors that affect depression, for example, one study found that the incidence of Major depressive disorder varies between urban and rural areas and between men and women in China (Gu et al., 2013). Meanwhile, education level is associated with depression, and some studies have found that people with lower levels of education are more likely to suffer from depression (Taple et al., 2022); we converted respondents’ education level into years of schooling in our regression analyses. The second group is the respondent’s socio-economic status, measured by several indications such as employment, and perceived income rank. The third dimension is the respondent’s health status, which mainly includes self-rated health, exercise, and hospitalization (see Table 1).

**Table 1:** Summary of the covariates.

Variables	Original question	Code
Gender	Respondent’s gender.	Male = 1, Female = 0.
Rural	The definition of urban and rural areas by the National Bureau of Statistics of China is used.	Rural = 1, others = 0.
Age	Current Age.	The variable takes the value of actual age.
Years of schooling	Your highest level of education?	We imputed the number of years of school based on the respondent’s education level.
Employment	Have you worked at least 1 hour in the past week?	Yes = 1, No = 0.
Perceived income rank	How would you rank your income locally?	1 for very low, 5 for very high.
Self-rated health	How do you rate your health at present?	The options are: 1. excellent, 2. very healthy, 3. relatively healthy, 4. average, 5. unhealthy; each answer was coded as 5, 4, 3, 2, or 1.
Exercises	Excluding biking and walking to and from work, how often did you participate in physical fitness and leisure activities in the past 12 months?	The options are 1. less than 1 time per month, 2. more than 1 time per month, but less than 1 time per week, 3. 1–2 times per week, 4. 3–4 times per week, 5. 5 times per week and more, 6. 1 time per day, 7. twice per day and more, 8. never attends. Each answer was coded as 1, 2, 3, 4, 5, 6, 7, 0.
Hospitalization	Have you been hospitalized for a medical condition in the past 12 months.	Yes is 1, No is 0.

### 2.5 Research hypothesis and methodology

The central question of our study is the impact of the left-behind experience on the mental health of left-behind spouses. Based on existing research, we formulated the following hypothesis: the left-behind experience will negatively impact the mental health of left-behind spouses, the strength of this effect varies between groups. To test this hypothesis, we conducted the following analyses separately. First, we describe the variables used in the regression to visualize the effect of spousal migration on the mental health and marital satisfaction of left-behind spouses. Second, taking the overall sample as the scope, we use regression equations to analyze the effects of long-term, short-term, and previous left-behind experiences on left-behind spouses’ depression and marital satisfaction. Depression and marital satisfaction are the dependent variables in the regression equations, whereas the duration of left-behind experience is an independent variable, and the regression equation also includes control variables such as age, self-rated health, and so on. Finally, considering the great differences in the experience of staying behind among different groups, we further conducted regression analyses with urban and rural, male and female samples to analyze the urban–rural and male–female differences in the impact of spousal migration on the mental health and marital satisfaction of left-behind spouses. We use R for statistical analysis, which is a free and efficient statistical software.

## 3 Results

### 3.1 Description of variables

Based on respondents’ answers about whether their spouses were away from home between 2020 and 2022, we categorized respondents with left-behind experiences into three groups. Eighty-nine respondents have spouses who do not live at home in both the 2020 and 2022 surveys, which we refer to as respondents with long-term left-behind experience; 193 respondents have spouses who live at home in 2020 and are not at home in 2022, which we refer to as respondents with short-term left-behind experiences; and 155 respondents have spouses who work away from home in 2020 and are back home in 2022, which we refer to as respondents with former left-behind experiences. The descriptive values of the variables for these three groups are shown in Table 2 below.

**Table 2:** Descriptive statistics of the variables.

Variable	Overall samples	Respondents with long-term left-behind experience	Respondents with short-term left-behind experience	Respondents with previous left-behind experience	Respondents lived in urban area	Respondents lived in rural area
Depression	13.78 (4)	15.62 (4.69)	14.65 (4.4)	14.35 (4.11)	13.48 (3.9)	14.15 (4.09)
Depression2020	13.41 (3.87)	14.45 (4.75)	14.07 (3.92)	14.25 (3.88)	13.12 (3.73)	13.75 (4)
Marital satisfaction	4.39 (0.89)	4.04 (1.16)	4.21 (1.06)	4.37 (0.84)	4.38 (0.87)	4.41 (0.91)
Marital satisfaction 2020	4.47 (0.83)	4.26 (1.04)	4.28 (1.03)	4.41 (0.8)	4.44 (0.84)	4.51 (0.83)
Gender	0.48 (0.5)	0.3 (0.46)	0.32 (0.47)	0.32 (0.47)	0.47 (0.5)	0.48 (0.5)
Rural	0.46 (0.5)	0.66 (0.48)	0.62 (0.49)	0.59 (0.49)	0	1
Age	44.75 (9.67)	41.85 (7.86)	42 (8.96)	42.46 (9.13)	43.95 (9.56)	45.68 (9.72)
Year of schooling	9.43 (3.89)	8.08 (3.76)	8.19 (3.42)	8.36 (3.6)	10.75 (3.85)	7.88 (3.33)
Employment	0.86 (0.35)	0.84 (0.37)	0.79 (0.41)	0.82 (0.39)	0.83 (0.37)	0.89 (0.31)
Perceived income rank	2.9 (0.99)	2.9 (1.12)	2.82 (1.02)	2.96 (1.06)	2.84 (0.94)	2.97 (1.04)
Self-rated health	3.15 (1.13)	3.16 (1.28)	3.03 (1.12)	3.14 (1.2)	3.14 (1.06)	3.15 (1.21)
Hospitalization	0.09 (0.29)	0.16 (0.37)	0.12 (0.32)	0.1 (0.3)	0.09 (0.28)	0.09 (0.29)
Exercise	1.53 (2.23)	1.28 (2.13)	0.98 (1.86)	1.45 (2.21)	1.88 (2.32)	1.12 (2.05)
N	8,096	89	193	155	4,375	3,721

Data shown are means and standard deviations.

It can be seen that, in terms of the depression value, the respondents with long-term left-behind experience have the highest depression value of 15.62, much higher than the overall sample mean of 13.78, while the respondents with short-term left-behind experience also have a higher depression value of 14.65. The mean depression value for respondents whose spouses had returned home was 14.35, reflecting the persistence of the impact of spousal leaving on respondents’ mental health, and even when spouses returned home, the depression value remained higher for respondents who had been reunited. In terms of marital satisfaction, the overall sample had a marital satisfaction value of 4.39, the respondents with long-term left-behind experience have the lowest marital satisfaction value of 4.04, while the respondents with short-term left-behind experience have a lower marital satisfaction value of 4.21, and the former stayer have a higher marital satisfaction value of 4.37. In addition, rural respondents had poorer mental health than urban respondents (14.15 vs. 13.48), but rural respondents had higher marital satisfaction than urban respondents (4.41 vs. 4.38).

Among the other variables, we can see that in the gender variable, the proportion of males in the total sample is 48%. However, the proportion of females among the left-behind respondents is comparatively higher, reflecting the fact that most of the time, males are out of the house for work, and females stay at home. In terms of the urban–rural distribution variable, 46% of the overall sample lived in the countryside, while 66% of those who had the long-term experience of staying behind lived in rural areas. In terms of age, most of the respondents with the experience of being left behind are younger than the average age of the overall sample; in terms of education level, the average education level of the respondents with the experience of being left behind is lower. In addition, in terms of physical exercise indicators, we can find that the frequency of physical exercise is lower among the respondents with the experience of being left behind, reflecting the fact that when their spouses work away from home, those who are left behind at home need to take care of the family and have less time at their disposal, thus reducing the frequency of physical exercise.

Since the data in Table 2 shows that there are significant differences in depression values and marital satisfaction between males and females and between urban and rural areas, we next present the depression and marital satisfaction for different age groups in 2022. Table 3 shows the mean values of depression and marital satisfaction for different age groups. As can be seen from the table, men aged 24–35 have the lowest average depression values of the three age groups; this result is consistent with the findings of a previous study, which found that the prevalence of depression in industrialized countries peaks in middle age (Bilu et al., 2023). The depression value of men in the same age group is generally lower than that of women; this is consistent with existing research (Albert, 2015). The fact that men are more satisfied with their marriages than women is consistent with the findings of many studies, such as one that found that husbands are more satisfied with their marriages than wives (Lavner and Bradbury, 2010). The mental health status of urban residents in the same age group is also generally better than that of rural residents, with some scholars suggesting that socioeconomic status is a major factor in the urban–rural depression gap (Li et al., 2016).

**Table 3:** Data on depression values and marital satisfaction for different age groups.

Variable	Depression	Marital satisfaction	*N*
Male Respondents lived in urban (24–35)	13 (3.58)	4.64 (0.69)	467
Female Respondents lived in urban (22–35)	14.04 (3.85)	4.17 (0.88)	652
Male Respondents lived in rural (24–35)	12.97 (3.49)	4.67 (0.72)	323
Female Respondents lived in rural (22–35)	14.32 (3.65)	4.17 (0.94)	452
Male Respondents lived in urban (36–45)	13.46 (3.78)	4.57 (0.76)	609
Female Respondents lived in urban (36–45)	13.46 (3.8)	4.17 (0.89)	626
Male Respondents lived in rural (36–45)	13.71 (3.89)	4.58 (0.78)	458
Female Respondents lived in rural (36–45)	14.4 (3.76)	4.26 (0.95)	479
Male Respondents lived in urban (46–60)	13.16 (3.98)	4.56 (0.8)	996
Female Respondents lived in urban (46–60)	13.66 (4.07)	4.23 (0.95)	1,025
Male Respondents lived in rural (46–60)	13.66 (4.2)	4.55 (0.84)	1,004
Female Respondents lived in rural (46–60)	15.01 (4.38)	4.3 (1)	1,005

Data shown are means and standard deviations.

Table 4 shows data on the gender and urban/rural distribution of respondents with experience of staying behind; of the total sample of 437 with experience of staying behind, 68.6% were female, and 61.8% were rural residents, compared with 52% female and 46% rural residents in the overall sample. Given the disproportionate distribution of left-behind experiences among women and rural areas and the reality that there are differences in mental health influences between men and women, and that the majority of internal migrants in China migrate from rural to urban areas, this suggests that it makes sense to proceed with a sub-sample regression according to gender and rural/urban areas.

**Table 4:** Gender and rural/urban distribution of those with the left-behind experience.

Groups	Overall samples	Respondents with long-term left-behind experience	Respondents with short-term left-behind experience	Respondents with previous left-behind experience
Male	137	27	61	49
Female	300	62	132	106
Urban	167	30	74	63
Rural	270	59	119	92

### 3.2 The impact of left-behind experience on depression

In order to study the impact of the left-behind experience on the mental health, five multiple regression equations were used for validation. In the first model, we use the overall sample for regression; in the second model, we use the male sample for regression, in the third model, we use the female sample for regression; in the fourth model, we use the urban sample for regression; and in the fifth model, we use the rural sample for regression. Preliminary analysis showed that the data did not violate the assumptions of the regression analysis. The regression results are displayed in Table 5.

**Table 5:** Regression results of the impact of left-behind experience on depressive symptoms.

Variables	Model 1 (Overall)	Model 2 (Male)	Model 3 (Female)	Model 4 (Urban)	Model 5 (Rural)
Depression 2020	0.46*** (0.01)	0.46*** (0.01)	0.46*** (0.01)	0.48*** (0.01)	0.44*** (0.01)
Short-term left-behind experience	0.25 (0.25)	0.43 (0.43)	0.12 (0.3)	0.68* (0.38)	0.01 (0.32)
Previous left-behind experience	0.06 (0.27)	−0.78 (0.48)	0.41 (0.33)	0.15 (0.42)	0.01 (0.36)
Long-term left-behind experience	1.16*** (0.36)	0.09 (0.65)	1.54*** (0.43)	−0.007 (0.6)	1.71*** (0.45)
Ref: No left-behind experience					
Age	−0.02*** (0.004)	−0.008 (0.006)	−0.03*** (0.006)	−0.03*** (0.006)	−0.01 (0.01)
Years of schooling	−0.08*** (0.01)	−0.043*** (0.016)	−0.11*** (0.015)	−0.05*** (0.01)	−0.11*** (0.02)
Employment	−0.36*** (0.11)	−0.81*** (0.25)	−0.17 (0.13)	−0.31** (0.14)	−0.62*** (0.18)
Perceived income rank	−0.24*** (0.04)	−0.27*** (0.06)	−0.23*** (0.05)	−0.32*** (0.05)	−0.2*** (0.06)
Self-rated health	−0.57*** (0.04)	−0.51*** (0.05)	−0.6*** (0.05)	−0.54*** (0.05)	−0.57*** (0.05)
Hospitalization	0.33** (0.13)	0.32 (0.2)	0.3* (0.18)	0.19 (0.18)	0.48** (0.2)
Exercise	−0.11*** (0.02)	−0.08*** (0.03)	−0.13*** (0.02)	−0.12*** (0.02)	−0.07** (0.03)
*N*	8,096	3,857	4,239	4,375	3,721
Adj *R*-squared	0.2989	0.274	0.312	0.2998	0.2941

*, *p* < 0.1; **, *p* < 0.05; ***, *p* < 0.01.

The regression results of Model 1 for the overall sample show that short-term left-behind experience and previous left-behind experience have no significant effect on mental health, and only long-term left-behind experience has a significant negative impact on mental health, leading to an increase in depression value by 1.16. The F-statistic for model 1 is 314.8 with a *p*-value < 0.0001, indicating that the overall design of our linear model is statistically significant; we also calculated the variance inflation factor (VIF) value for each variable in model 1, and the VIF values for each variable range from a low of 1.01 to a high of 1.3, which is much less than 5, indicating that there is no multicollinearity problem in the model design. Model 2 reveals that spouses’ departure from home does not have significant effects on the psychological health of males, while Model 3 shows that, in the case of females, spouses’ departure from home during both surveys leads to an increase in the depression value of left-behind wives by 1.54. Model 4 shows that for the urban sample, short-term left-behind experience leads to an increase of depression value; model 5 shows that in the rural sample, long-term left-behind experience leads to an increase of 1.71 in the depression value of the left-behind spouse. The regression results of models 2, 3, 4, and 5 show that the p-value of the F-statistic values is less than 0.0001, indicating that the model is well-designed, and the VIF test found that the VIF coefficients of all the variables are between 1 and 2, indicating that multicollinearity is not a problem. From the above regression results, we believe that our hypothesis is validated.

Among the covariates, broadly speaking, variables such as age, years of schooling, employment, perceived income level, exercise, and self-rated health were negatively correlated with depression values, while variables such as hospitalization within 1 year, which represents physical health, were positively correlated with depression values, suggesting that the more unhealthy one is, the worse one’s mental health status is, and these findings are consistent with the results of the majority of the existing studies.

### 3.3 The impact of left-behind experience on marital satisfaction

To study the impact of the left-behind experience on marital satisfaction, we also used five multiple regression equations for validation. In the first model, we use the overall sample for regression; in the second model, we use the male sample for regression; in the third model, we use the female sample for regression; in the fourth model, we use the urban sample for regression; and in the fifth model, we use the rural sample for regression. Preliminary analysis showed that the data did not violate the assumptions of the regression analysis. The regression results are all displayed in Table 6.

**Table 6:** Regression results of the impact of left-behind experience on marital satisfaction.

Variables	Model 1 (Overall)	Model 2 (Male)	Model 3 (Female)	Model 4 (Urban)	Model 5 (Rural)
Marital satisfaction 2020	0.44*** (0.01)	0.4*** (0.02)	0.42*** (0.01)	0.46*** (0.01)	0.42*** (0.02)
Short-term left-behind experience	−0.09 (0.06)	0.13 (0.09)	−0.16** (0.07)	−0.2** (0.09)	−0.02 (0.08)
Previous left-behind experience	−0.001 (0.064)	−0.08 (0.1)	0.07(0.08)	−0.05 (0.1)	0.03 (0.09)
Long-term left-behind experience	−0.26*** (0.08)	−0.26* (0.14)	−0.23** (0.11)	−0.18 (0.14)	−0.3*** (0.11)
Ref: No left-behind experience					
Covariances	Y	Y	Y	Y	Y
*N*	8,096	3,857	4,239	4,375	3,721
Adj R-squared	0.2148	0.1565	0.2218	0.2417	0.1869

*, *p* < 0.1; **, *p* < 0.05; ***, *p* < 0.01.

The regression results of Model 1 show that the left-behind experience affects the marital satisfaction of left-behind spouses, but this effect is heterogeneous; short-term left-behind experience and previous left-behind experience do not have a significant impact on left-behind spouses’ marital satisfaction. However, the long-term left-behind experience had a significant negative impact on marital satisfaction, resulting in a decrease of 0.26 in marital satisfaction. The *F*-statistic for model 1 is 202.3 with a *p*-value < 0.0001, indicating that the overall design of our linear model is statistically significant; we also calculated the VIF value for each variable in model 1, and the VIF values for each variable range from a low of 1.01 to a high of 1.28, indicating that there is no multicollinearity problem in the model design. The regression results of Model 2 show that the long-term left-behind experience decreases marital satisfaction for male left-behind spouses. Model 3 shows that for women, both short-term and long-term left-behind experience decreases marital satisfaction; Model 4 shows that for urban respondents, short-term left-behind experience decreases marital satisfaction while long-term left-behind experience does not affect marital satisfaction; Model 5 shows that for rural respondents, long-term left-behind experience significantly decreases marital satisfaction by 0.3. The results of models 2, 3, 4, and 5 show that the p-value of the F-statistic values is less than 0.0001, indicating that the model is well-designed, and the VIF test found that the VIF coefficients of all the variables are between 1 and 2, indicating that multicollinearity is not a problem.

## 4 Discussion

Firstly, the short-term left-behind experience is less likely to affect the mental health of the left-behind spouse, a point not made in many studies. As far as we know, there is a study that states that short-term separation does not necessarily lead to deterioration of mental health (Oblea et al., 2016). As for the reason why short-term separation did not affect mental health. It may be that the sample in our study is younger, the financial pressures of supporting their children and older parents are higher, and it is more likely that the spouse’s departure from the home to earn money to support the family brings better financial income to the family (Silvey, 2006), and the income effect is sufficient to compensate for the pain of separation, thus cushioning the mental health impact of the spouse’s migration away from home to work. Some of those who stayed behind said in interviews, it is better to earn more money separately than to suffer poverty together (Biao, 2007). Therefore, the short-term left-behind experience is usually not associated with significant mental health impacts.

Secondly, the long-term left-behind experience leads to poor mental health, which is consistent with many studies (Siriwardhana et al., 2015). For example, a study based on Chinese data found that left-behind spouses had higher depression scores compared to rural residents with intact families (Nikoloski et al., 2019). The results of a study showed that more than one-third of rural left-behind women in northwestern China had depressive symptoms (35.7%) (Niu and Wang, 2024). An Indonesian study also found that left-behind adults are more prone to psychological distress (Lu, 2012). Our study also found that the negative impact of the left-behind experience on mental health was particularly pronounced in the rural resident and female samples; this is consistent with findings in China and other countries. For example, a study in China found that left-behind status was an independent risk factor for depression in rural women (Jin et al., 2016). A study in Tajikistan found higher rates of depression among migrant wives (Pirova et al., 2018). The impact of the left-behind experience on mental health may be due to the lack of spousal support and the added psychological stress of caring for children and families (Roy and Nangia, 2005). Left-behind mothers face particular pressures and loneliness (Nguyen et al., 2024). At the same time, women who stay behind have poorer sleeping conditions (Wang et al., 2021). Moreover, the spatial separation of couples leads to uncertainty about the future, which in turn leads to loneliness, sadness, and possible alienation (Samokhvalova et al., 2022).

Thirdly, the transition from staying behind to reunification did not result in a decrease in the depression value, suggesting that the shock factor of spouses working outside the home has a profound impact on the mental health of individuals. This finding is consistent with many studies. For example, one study found that the prevalence of obsessive compulsion among college students with left-behind experience was 44.05%, which was higher than the control group’s 28.56% (Liu et al., 2020). The stay-behind experience is akin to a life crisis, and it takes time to recover from the effects of such a crisis on mental health. There is an idea that chronic stress, such as bad life experiences in early life, may have lasting impact on the brain and body, even if they disappear (McEwen, 2017). Therefore, past experiences of staying behind often constitute a chronic stressor; even if one no longer stays behind, it takes time to recover from the mental health damage caused by past experiences of staying behind.

Finally, short-term and long-term left-behind experiences can lead to declining marital satisfaction, but this effect is also heterogeneous. For both the urban and female samples, short-term left-behind experiences weaken marital satisfaction. This is a relatively novel finding, and there is little literature pointing this out. In addition, long-term left-behind experience leads to lower marital satisfaction, except in urban samples. The impact of the stay-behind experience on marital satisfaction may be due to the inability of stay-behind spouses to receive face-to-face emotional support from their spouses, and emotional support from spouse has a significant impact on marital satisfaction (Iwasa et al., 2024). Also, there are significant differences in the factors that affect marital satisfaction between men and women; for example, one study found that female respondents identified communication, understanding, and in-law relationships as factors that affected their marital satisfaction (Ayub and Iqbal, 2012).

## 5 Conclusion

Our study found that spouses going out to work affects the mental health and marital satisfaction of left-behind spouses, which is consistent with the results of some studies. However, the effects of long-term and short-term left-behind experiences on mental health and marital satisfaction were inconsistent, with long-term left-behind experiences impairing the mental health and marital satisfaction of left-behind spouses. In contrast, short-term left-behind experiences did not affect mental health, but short-term left-behind experiences had a heterogeneous effect on marital satisfaction. After confirming the negative impact of the left-behind experience on mental health and marital satisfaction, to improve people’s sense of well-being, we believe that efforts should be made to reduce the phenomenon of spousal separation due to going out to work and to promote more local employment; at the same time, our study shows that rural left-behind women are an especially disadvantaged group and that efforts should be made to improve their mental health and to provide them with more social support and psychological interventions.

## 6 Recommendation

Based on the results of our study. We make the following recommendations. Firstly, given that the experience of staying behind can negatively affect a person’s mental health and marital satisfaction, the state should formulate policies to minimize separation due to work and employment, for example, by promoting collaborative regional development, by vigorously fostering economic growth in the places where the labor force leaves, by promoting local employment. Secondly, our previous study found that short-term left-behind experiences were not significantly detrimental to mental health. So, spouses who have left home should minimize the time they spend away from home to shorten the time left behind by their spouses. At the same time, during the period away from home, they should improve their exchanges and communication with their spouses, pay more attention to the health of their spouses, and provide their spouses with more spiritual solace. Thirdly, attention should be focused on the mental health of female left-behind spouses in rural areas, who are a very vulnerable group in terms of mental health; China has formulated the "Action Program for Precise Care and Support for Difficult Groups of Rural Left-behind Women” in 2025. According to the program, more public cultural services and diversified support will be provided to rural left-behind women in the future. We suggest that the needs of rural left-behind women should be accurately grasped at the community level and that differentiated public services and support should be provided.

## 7 Limitations

To the best of our knowledge, this is one of the few literatures that study the mental health and marital satisfaction of left-behind spouses based on Chinese data, and our article makes a marginal contribution to this field. However, our paper has some limitations. First, although we used data from 2020 and 2022, our analysis is still essentially a cross-sectional analysis, which can yield statistical relationships between the data but not causality; second, we used a public data set that contains many variables, and this survey lacks data on the emotional exchanges and interactions between the left-behind spouses and their spouses, and thus does not more accurately capture the factors that affecting the psychological health and marital satisfaction of left-behind spouses; third, although we used the concepts of long-term left-behind and short-term left-behind experiences, it is difficult to grasp the actual length of time that each respondent has stayed behind due to the limitations of the data, and therefore future research can be further developed based on clarifying the actual length of time that they have stayed behind. At the same time, we only used samples that participated in both the 2020 and 2022 surveys, deleting samples that had data from only a single year, which may have caused a selective bias that we could not grasp and the next step should be to conduct the study using a larger scale of data.

## Data Availability

Publicly available datasets were analyzed in this study. This data can be found at: https://www.isss.pku.edu.cn/cfps/index.htm.
